# The role of prenatal food insecurity on breastfeeding behaviors: findings from the United States pregnancy risk assessment monitoring system

**DOI:** 10.1186/s13006-020-00276-x

**Published:** 2020-04-19

**Authors:** Lauren M. Dinour, Elizabeth I. Rivera Rodas, Ndidiamaka N. Amutah-Onukagha, Laurén A. Doamekpor

**Affiliations:** 1 College of Education and Human Services, Montclair State University, 1 Normal Avenue, Montclair, NJ 07043 USA; 2 School of Medicine, Tufts University, 136 Harrison Avenue, Boston, MA 02111 USA; 3 Scientific Research, Health Policy Research Consortium, CTIS Inc, 6401 Golden Triangle Drive, Suite #310, Greenbelt, MD 20770 USA

**Keywords:** Breastfeeding initiation, Breastfeeding cessation, Food insecurity, PRAMS

## Abstract

**Background:**

In addition to its health and nutritional benefits, breastfeeding can save low-income, food insecure mothers the cost of infant formula so that money can be spent on food and other necessities. Yet breastfeeding may exacerbate food insecurity by negatively affecting maternal employment. The relationship between food insecurity and breastfeeding has been explored previously, with varying results. The purpose of this study was to determine the relationship between prenatal food insecurity and breastfeeding initiation and early cessation (< 10 weeks) among U.S. mothers.

**Methods:**

Data were pooled from 2012 to 2013 (Phase 7) of the Pregnancy Risk Assessment Monitoring System, a population-based cross-sectional survey of postpartum women administered 2–4 months after delivery. The analytic sample was drawn from Colorado, Maine, New Mexico, Oregon, Pennsylvania, and Vermont, and limited to mothers aged 20 years and older whose infants were alive and living with them at the time of the survey (*n* = 10,159). We used binomial and multinomial logistic models to assess the predictive association between food insecurity and breastfeeding initiation and early cessation, respectively, while controlling for confounders.

**Results:**

Most women reported prenatal food security (90.5%) and breastfeeding initiation (91.0%). Of those who initiated breastfeeding, 72.7% breastfed for > 10 weeks. A larger proportion of food secure women compared to food insecure women, initiated breastfeeding (91.4% vs. 87.6%, *P* < 0.01), and patterns of early breastfeeding cessation differed significantly between the two groups (*P* < 0.01). In the final models, prenatal food insecurity was not associated with breastfeeding initiation or early cessation, with one exception. Compared to food secure mothers, mothers reporting food insecurity had a lower risk of breastfeeding for 4–6 weeks than for > 10 weeks, independent of covariates (relative risk ratio 0.65; 95% CI 0.50, 0.85; *P* < 0.01). Women who were married, had a college degree, and did not smoke were more likely to initiate breastfeeding and breastfeed for a longer time, regardless of food security status (*P* < 0.01).

**Conclusions:**

Socioeconomic, psychosocial, and physiological factors explain the association between prenatal food insecurity and breastfeeding outcomes among this U.S. sample. More targeted and effective interventions and policies are needed to encourage the initiation and duration of breastfeeding, regardless of food security status.

## Background

Breast milk is superior to infant formula, protecting infants from a host of infectious and chronic conditions [1–4]. Consequently, most national and international public health authorities recommend that infants be breastfed exclusively for the first 6 months of life, with continued breastfeeding alongside complementary foods through at least the first year or more [2, 4–6]. Even any breastfeeding confers health benefits compared to never breastfeeding [1, 3]. In the U.S., breastfeeding rates have improved over time [7] due to multi-pronged efforts aimed at education and policy changes. Yet many mothers, particularly low-income women, are not offered paid maternity leave and thus must return to work soon after birth [8, 9], making it difficult to establish a successful breastfeeding relationship. Likewise, employers may not fully support women who wish to express breast milk at work, and women may terminate breastfeeding as a result [10–13].

Food insecurity, the lack of enough money to purchase adequate amounts, variety, and quality of food, can act as a potential double burden for low-income women. When food insecurity is severe, there may be prolonged periods of disrupted eating patterns and reduced food intake [14]. Although the prevalence of food insecurity in the U.S. has declined in recent years (11.8% in 2017, down from 14.9% in 2011), risk remains substantially higher among households with incomes near or below the poverty line (30.8%) and among households with children headed by single women (30.3%) [14]. In theory, breastfeeding could save food insecure mothers the cost of formula so that money can be spent on food and other necessities. However, in countries like the U.S. without guaranteed paid maternity leave, adhering to breastfeeding recommendations may exacerbate food insecurity by preventing or negatively affecting maternal employment and earnings [15–17]. It is therefore important to understand the relationship between food insecurity and breastfeeding practices to provide every woman, regardless of income, with the true choice to breastfeed.

The relationship between food insecurity and breastfeeding behaviors has been assessed in several different geo-cultural contexts and appears complex. Among some less developed countries, such as Kenya [18] and Uganda [19], where HIV rates are relatively high, household food insecurity is not associated with maternal recall of exclusive breastfeeding or any breastfeeding at several time points after birth. Yet, greater household food insecurity is associated with a reduced volume of breast milk intake among infants [18], and mothers reporting moderate to severe household hunger are more likely than mothers experiencing little to no household hunger to cease exclusive breastfeeding between 4 and 6 months [19]. Additionally, food insecure mothers are significantly more likely than food secure mothers to believe exclusive breastfeeding for 6 months would be an insufficient mode of infant feeding, and that they would be unable to exclusively breastfeed for 6 months if recommended to do so by a healthcare worker [20]. Qualitative findings from Haiti paint a more nuanced relationship, whereby food insecurity among some mothers led to breastfeeding cessation due to perceived breast milk insufficiency and maternal weakness from undernutrition [21]. Conversely, some Haitian mothers reported breastfeeding continuation as a last resort in the absence of other foods or liquids [21].

Results vary in more developed countries, as well. Among a sample of Brazilian children under 2 years old, an association between breastfeeding and food insecurity was found for children between 12 and 24 months (but not younger than 12 months), whereby breastfeeding prevalence was higher among children living in food insecure households compared to food secure households [22]. In Canada, while household food insecurity is not related to breastfeeding initiation, the odds of exclusive breastfeeding at 4 months were significantly lower among women living in food insecure, compared to food secure, households [23]. Qualitative findings from Canada, however, suggest that food insecurity is a major contributor to breastfeeding initiation due to the worry over the cost of infant formula [24]. Additionally, food insecurity may be a root cause of breastfeeding cessation due to maternal fears of producing milk that is inadequate in quantity or quality [24]. In considering the reverse temporal pathway, Wong et al. found that breastfeeding duration does not predict household food insecurity [25]. Finally, among two samples of low-income, Hispanic or primarily Hispanic mother-infant pairs in New York City, neither partial nor exclusive breastfeeding was significantly associated with food insecurity, whether experienced prenatally, postnatally, or both [26, 27].

The lack of consistent findings is largely due to the variety of geographies, confounding circumstances (e.g., HIV status), breastfeeding outcome variables, food security measures, and research methods (e.g., qualitative and quantitative) used. Additionally, there is a need for representative studies from the U.S. that assess the relationship between food security and breastfeeding behaviors. The U.S. is unique in that it is the only developed country without a federally-mandated paid maternity leave [28], and it offers the Special Supplemental Nutrition Program for Women, Infants, and Children (WIC) [29] to provide low-income, at-risk participants with supplemental food, nutrition education, breastfeeding support, and infant formula. The purpose of the current study is to utilize the national Pregnancy Risk Assessment Monitoring System (PRAMS) dataset to determine if food insecurity is associated with breastfeeding initiation and early cessation (< 10 weeks) among U.S. mothers. PRAMS data is ideal for this research, as it surveys a U.S.-based representative sample of women during the first few months after birth and asks questions regarding both breastfeeding and household food security.

## Methods

### Data source and study population

This study pools cross-sectional PRAMS data from 2012 to 2013 (Phase 7) for Colorado, Maine, New Mexico, Oregon, Pennsylvania, and Vermont (*N* = 13,284). These states were chosen because they were the only ones with data on food insecurity, which is an optional question that states can choose to ask of their PRAMS respondents. However, it should be noted that the majority of these states exhibited higher than national averages for both breastfeeding initiation [7] and food security [30] (Table 1).

**Table 1:** Breastfeeding initiation and household food security prevalence nationally and among states included in this study

State	Breastfeeding Initiation Rate, 2013 (7)	Household Food Security Prevalence, Average 2011–2013 (21)
*U.S. National*	*81.1%*	*85.4%*
Colorado	88.6%	86.1%
Maine	86.6%	84.9%
New Mexico	85.5%	86.8%
Oregon	92.5%	84.8%
Pennsylvania	73.3%	88.1%
Vermont	84.5%	86.8%

**Fig. 1:**
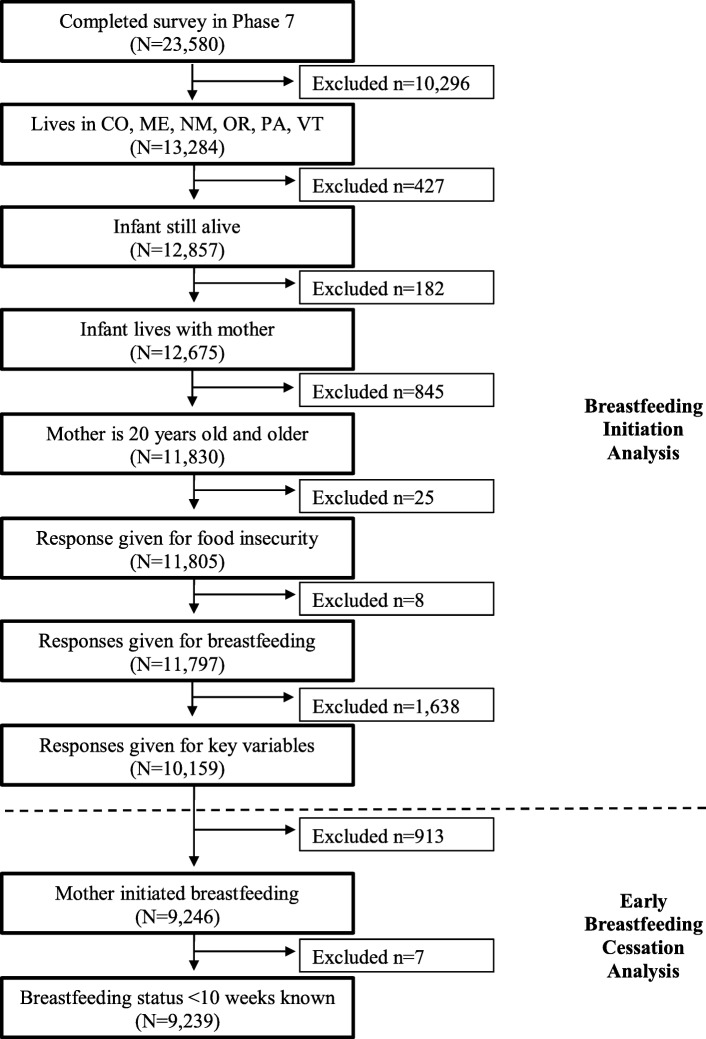
Flow diagram of sample selection for this study: PRAMS, United States, 2012–2013

PRAMS is a joint effort of the U.S. Center for Disease Control and Prevention (CDC) and state health departments and is a public health survey that uses standardized collection techniques to gather information from a random sample of resident women who delivered a live infant. PRAMS uses birth certificates as the sampling frame and identifies women who gave birth to a live infant within the previous 2–4 months. It then uses mailed questionnaires and telephone follow-ups to obtain information from a stratified representative sample of these women, with members of high-risk groups oversampled, and links questionnaire answers to birth certificate data. Additional details regarding the general methodology of PRAMS are available elsewhere [31]. For this study, the sample was limited to mothers aged 20 years and older whose infants were alive and living with them at the time of the survey (*n* = 11,830; Fig. 1).

### Human participant protection

The Montclair State University Institutional Review Board determined that no protocol approval was required because the study used secondary, de-identified data.

### Study variables

The independent variable was prenatal maternal food insecurity measured retrospectively at the time of the survey using the single standard question, “During the past 12 months before your new baby was born, did you ever eat less than you felt you should because there wasn’t enough money to buy food?” (yes/no). This standard question is one of the items included in the U.S. Department of Agriculture’s (USDA) abbreviated 6-item food security module [32] and has been used elsewhere [33, 34]. Responses were dichotomized to food insecure (yes) and food secure (no).

Two dependent variables were used: breastfeeding initiation and early breastfeeding cessation. To assess breastfeeding initiation, PRAMS asks the following question: “Did you ever breastfeed or pump breast milk to feed your new baby, even for a short period of time?” (yes/no). Responses were dichotomized as mothers who did initiate breastfeeding (yes) and those who did not (no). To determine early cessation among respondents who initiated breastfeeding, the number of weeks of breastfeeding was measured using the following survey questions: “Are you currently breastfeeding or feeding pumped milk to your new baby?” (yes/no) and if no, “How many weeks or months did you breastfeed or pump milk to feed your baby?” For mothers who were still breastfeeding at the time of the survey, we determined the age of the infant in days and removed respondents whose infants were less than 10 weeks (70 days) old (*n* = 7). We chose 10 weeks as the cut-point to maximize our sample size, since all but seven respondents who were still breastfeeding at the time of the survey completed the survey when the infant was 10 weeks old or older. Later cut-points, such as 4 months, would have removed over half of the sample. Additionally, much of the prior breastfeeding research based on PRAMS data utilizes the 10-week cut-point in an effort to give all respondents an equal opportunity for inclusion in the analysis [35–39]. The early breastfeeding cessation variable was classified into five categories (< 1 week, 1–3 weeks, 4–6 weeks, 7–9 weeks, and > 10 weeks), with those still breastfeeding at the time of the survey classified as > 10 weeks and those who did not initiate breastfeeding excluded from analysis. Exclusive breastfeeding could not be determined as PRAMS does not ask about the feeding of anything other than breastmilk.

Potentially confounding variables included in our multivariable analysis were identified from previous studies of food insecurity and breastfeeding. Of particular note, Webb-Girard et al. [20] posited multiple mechanisms by which food insecurity may adversely affect exclusive breastfeeding. These include socio-economic factors, psychosocial factors, and physiological factors. For example, with regards to socio-economic factors, food insecurity may lead women to seek employment outside of the home, which can negatively impact breastfeeding behaviors. Psychosocially, the distress and depression caused by food insecurity may undermine women’s confidence and self-efficacy to breastfeed. The stress associated with food insecurity may also physiologically impair milk output, and thus reduce the likelihood of exclusive breastfeeding [20]. For our study, we grouped potentially confounding variables using similar categories presented by Webb-Girard et al.’s [20] conceptual framework: sociodemographic, psychosocial, and physiological variables. Sociodemographic variables included maternal age in years (20–24; 25–29; 30–34; > 35), annual income (< $22,000; $22,001–$37,000; $37,001–$52,000; $52,001–$67,000; > $67,001), self-reported marital status (married; not married), years of maternal education (< 12 years; 12 years; 13–15 years, > 16 years), race/ethnicity (Hispanic of any race; non-Hispanic White; non-Hispanic Asian/Pacific Islander; non-Hispanic Black; non-Hispanic American Indian/Alaska Native; non-Hispanic other or mixed race; unknown race and/or ethnicity), educational information regarding breastfeeding provided by a healthcare worker (received breastfeeding information; did not receive breastfeeding information), and receipt of WIC during pregnancy (received WIC; did not receive WIC). Medical insurance at the time of the survey was categorized as Government (Medicaid; Medicare; Children’s Health Insurance Program (CHIP)/State CHIP; or other governmental insurance), Private (insurance paid by an employer; purchased from a company; or TRICARE/military insurance), Other (insurance from some other source), and None (no insurance or insurance through the Indian Health Service). These insurance categories were based on prior research analyzing PRAMS data [40].

Psychosocial variables included postpartum depression (yes/no), total number of stresses in the 12 months before birth (a continuous variable between 0 and 14 based on a sum of positive responses to 14 possible stressful events that have happened during the 12 months before the new baby was born such as moving, divorce, loss of a job, or a death), and whether the mother wanted to be pregnant (later; sooner; then; never; unsure). Physiological variables included delivery method (vaginal delivery; non-vaginal delivery), the Kotelchuck index for prenatal care (inadequate; intermediate; adequate; adequate plus), infant’s length of hospital stay (0–2 days; 3–5 days; > 6 days), and mother’s smoking status at the time of the survey (smoker; non-smoker).

Potentially confounding variables were collected by self-report from mothers via the PRAMS survey, except for maternal age, marital status, maternal education, maternal race/ethnicity, delivery method, and Kotelchuck Index, which originated from the birth certificate. Additional potentially confounding variables were considered but not included in the final analysis, either because there were values for only a small percentage of the sample (i.e., trying to get pregnant, or already pregnant, at the time of the survey), issues with multicollinearity (i.e., maternal pre-pregnancy body mass index), or non-significant relationships in binomial logistic regression models that predicted breastfeeding initiation or early cessation (i.e., plurality, birth order, gestational age, birthweight, birth defect, infant in intensive care at birth, and maternal medical risk factors).

### Statistical analysis

All analyses were conducted with survey procedures in STATA MP version 16 and the standard errors adjusted for the complex sampling survey design. PRAMS’ weighted variables were used to account for the PRAMS survey design and the statistical weighting of the data. The analytic sample was limited to women with data for both food security status and breastfeeding outcomes (*n* = 10,159, Fig. 1). We assessed the sample by food security status and conducted chi-square tests and t-tests to examine the distribution of each variable. To assess the best predictive association between prenatal food insecurity and breastfeeding initiation, we used binomial logistic models and report the odds ratios (OR). Model 1 included prenatal food security status to assess the unadjusted relationship between the independent and dependent variables. Model 2 added socioeconomic status variables: maternal age, income, marital status, maternal education, maternal race/ethnicity, insurance type at the time of survey, breastfeeding information provided by a healthcare provider, and WIC status during pregnancy. Model 3 added psychosocial variables: postpartum depression, number of stresses during pregnancy, and pregnancy intention. Model 4 included physiological variables: delivery method, Kotelchuck index, length of hospital stay, and current smoking status.

Multinomial logistic models were used to assess the best predictive association between prenatal food insecurity and early breastfeeding cessation among respondents who initiated breastfeeding (*n* = 9239). From these models, we present the relative risk ratios (RRR) comparing breastfeeding durations of < 1 week, 1–3 weeks, 4–6 weeks, and 7–9 weeks to the reference of breastfeeding for > 10 weeks. Four models were also conducted for the multinomial logistic models where Model 1 assessed the relationship between prenatal food insecurity and early breastfeeding cessation without controlling for any potential confounders. Model 2 added socioeconomic status variables, Model 3 added psychosocial variables, and Model 4 added physiological variables.

The traditional *P* < 0.05 criterion of statistical significance was employed for all tests within each model. However, in order to avoid inflated likelihood of error in the multinomial logistic models, we use an adjusted *p* - value to test for significance between models. To calculate this using Bonferroni’s method, we divided our original *p* - value of 0.05 by the number of models (4), giving us a new threshold of significance (*P* < 0.0125). For simplicity, we use *P* < 0.01 which maintains our 95% confidence in our set of analyses as a whole.

## Results

Table 2 describes the sample. A total of 10,159 women were included in the final analysis, most of whom were 25–29 years old (31.1%), non-Hispanic White (69.9%), married (66.8%), and had at least some college education (68.2%). Thirty-three percent of women had an annual income of $22,000 or below and 29.6% made more than $67,000 in the 12 months before giving birth. A little over half of the sample had private insurance (54.9%) and 39.9% of women were receiving WIC benefits during pregnancy. Nearly 80% of the sample received adequate or adequate plus prenatal care, 85.1% had a prebirth conversation with a healthcare worker about breastfeeding, 10.9% reported experiencing postpartum depression, and 15.2% smoked at the time of the survey. Differences were found between food security groups for most socioeconomic, psychosocial, and physiological characteristics (Table 2).

**Table 2:** Maternal characteristics, overall and by prenatal food security status

Characteristics	Overall (***N*** = 10, 159)	Food Secure (***n*** = 9, 190)	Food Insecure (***n*** = 969)	T-test or χ^**2**^***P***-value
Breastfeeding initiation, No. (%)	9246 (91.01)	8397 (91.37)	849 (87.62)	0.00
Early breastfeeding cessation, No. (%)^a^				
< 1 week	241 (2.61)	203 (2.42)	38 (4.48)	0.00
1–3 weeks	747 (8.09)	644 (7.68)	103 (12.13)	
4–6 weeks	848 (9.18)	761 (9.07)	87 (10.25)	
7–9 weeks	687 (7.44)	588 (7.01)	99 (11.66)	
≥ 10 weeks	6716 (72.69)	6194 (73.83)	522 (61.48)	
Grouped maternal age, No. (%)				
20–24 years old	2279 (22.43)	1884 (20.50)	395 (40.76)	0.00
25–29 years old	3158 (31.09)	2882 (31.36)	276 (28.48)	
30–34 years old	3021 (29.74)	2830 (30.79)	191 (19.71)	
35+ years old	1701 (16.74)	1594 (17.34)	107 (11.04)	
Income 12 months before, No. (%)				
$0–$22,000	3390 (33.37)	2736 (29.77)	654 (67.49)	0.00
$22,001-37,000	1685 (16.59)	1487 (16.18)	198 (20.43)	
$37,001-52,000	1178 (11.60)	1096 (11.93)	82 (8.46)	
$52,001-67,000	900 (8.86)	878 (9.55)	22 (2.27)	
$67,001+	3006 (29.59)	2993 (32.57)	13 (1.34)	
Marital status, No. (%)				
Not married	3372 (33.19)	2815 (30.63)	557 (57.48)	0.00
Married	6787 (66.81)	6375 (69.37)	412 (42.52)	
Years of maternal education, No. (%)				
0–11 years	971 (9.56)	823 (8.96)	148 (15.27)	0.00
12 years	2261 (22.26)	1929 (20.99)	332 (34.26)	
13–15 years	3105 (30.56)	2724 (29.64)	381 (39.32)	
16+ years	3822 (37.62)	3714 (40.41)	108 (11.15)	
Race/ethnicity, No. (%)				
Hispanic	1283 (12.63)	1151 (12.52)	132 (13.62)	0.33
NH White	7098 (69.87)	6483 (70.54)	615 (63.47)	0.00
NH Black	402 (3.96)	338 (3.68)	64 (6.60)	0.00
NH Asian	416 (4.09)	399 (4.34)	17 (1.75)	0.00
NH Native American	374 (3.68)	310 (3.37)	64 (6.60)	0.00
NH Other	422 (4.15)	358 (3.90)	64 (6.60)	0.00
Unknown race/ethnicity	164 (1.61)	151 (1.64)	13 (1.34)	0.48
Insurance type, No. (%)				
Government insurance	3088 (30.40)	2561 (27.87)	527 (54.39)	0.00
Private insurance	5578 (54.91)	5357 (58.29)	221 (22.81)	0.00
Other insurance	149 (1.47)	132 (1.44)	17 (1.75)	0.43
No insurance	1344 (13.23)	1140 (12.40)	204 (21.05)	0.00
Healthcare worker talked about breastfeeding before birth, No. (%)	8641 (85.06)	7808 (84.96)	833 (85.96)	0.40
On WIC during pregnancy, No. (%)	4053 (39.90)	3369 (36.66)	684 (70.59)	0.00
Experienced postpartum depression, No. (%)	1111 (10.94)	856 (9.31)	255 (26.32)	0.00
Number of stresses in 12 months before birth, mean (SD)	1.99 (2.02)	1.74 (1.81)	4.32 (2.42)	0.00
Wanted to be pregnant, No. (%)				
Later	2061 (20.29)	1779 (19.36)	282 (29.10)	0.00
Sooner	1513 (14.89)	1416 (15.41)	97 (10.01)	0.00
Then	4665 (45.92)	4398 (47.86)	267 (27.55)	0.00
Never	591 (5.82)	479 (5.21)	112 (11.56)	0.00
Unsure	1329 (13.08)	1118 (12.17)	211 (21.78)	0.00
Vaginal delivery, No. (%)	6774 (66.68)	6119 (66.58)	655 (67.60)	0.52
Kotelchuck Index, No. (%)				
Inadequate	1028 (10.12)	857 (9.33)	171 (17.65)	0.00
Intermediate	1175 (11.57)	1067 (11.61)	108 (11.15)	
Adequate	4441 (43.71)	4070 (44.29)	371 (38.29)	
Adequate Plus	3515 (34.60)	3196 (34.78)	319 (32.92)	
Hospital baby stay length grouped, No. (%)				
0–2 days	5967 (58.74)	5439 (59.18)	528 (54.49)	0.02
3–5 days	2698 (26.56)	2419 (26.32)	279 (28.79)	
6+ days	1494 (14.71)	1332 (14.49)	162 (16.72)	
Smoking at time of survey, No. (%)	1526 (15.20)	1176 (12.80)	350 (36.12)	0.00

*Note.NH* non-Hispanic, *SD* standard deviation, *WIC* Special Supplemental Nutrition Program for Women, Infants, and Children; ^a^Early breastfeeding cessation analysis excludes respondents who did not initiate breastfeeding (*n* = 913) and those who were still breastfeeding at the time of the survey but completed the survey before 10 weeks post birth (*n* = 7). Thus, the early breastfeeding cessation sample size is N = 9239 for overall, *n* = 8390 for food secure, and *n* = 849 for food insecure. Percentages within this category are calculated based on these sample sizes

The majority of the sample reported they were food secure during the 12 months prior to birth (90.5%) and initiated breastfeeding (91.0%). Among those who initiated breastfeeding, 72.7% of the sample breastfed for > 10 weeks. A larger proportion of food secure women compared to food insecure women, initiated breastfeeding (91.4% vs. 87.6%, *P* < 0.01). Patterns of early breastfeeding cessation were also significantly different between the two groups (*P* < 0.01; Table 2). Notably, among food secure women, the highest percentage of early breastfeeding cessation occurred during 4–6 weeks (9.1%), whereas the largest percentage of food insecure women stopped breastfeeding between 1 and 3 weeks (12.2%), followed by 7–9 weeks (11.7%).

### Breastfeeding initiation

In the unadjusted multivariate model, women who were food insecure in the 12 months prior to birth were less likely to have ever breastfed compared to women who were food secure (OR 0.67; 95% Confidence Interval [CI] 0.54, 0.82; Table 3). However, this effect disappeared when the potentially confounding variables were included. Among the sociodemographic variables, all but income were significantly associated with breastfeeding initiation in the final model (Model 4). Specifically, compared to older women (> 35 years), those between the ages of 20–24 were about 1.5 times more likely to initiate breastfeeding (OR 1.45; 95% CI 1.12, 1.88). Married women had a greater odds of initiating breastfeeding compared to their unmarried counterparts (OR 1.29; 95% CI 1.09, 1.54). Less educated women were less likely to initiate breastfeeding compared to those with 16+ years of education. Of note, women with some college (13–15 years of education) were nearly half as likely (OR 0.53; 95% CI 0.41, 0.67), and those with < 12 years about 70% less likely, to initiate breastfeeding compared to their more educated counterparts.

**Table 3:** Results of binomial logistic regression predicting breastfeeding initiation

	Model 1, OR (95% CI)	Model 2, OR (95% CI)	Model 3, OR (95% CI)	Model 4, OR (95% CI)
Food insecurity				
Food secure (Ref.)	1.00	1.00	1.00	1.00
Food insecure	0.67** (0.54, 0.82)	1.09 (0.88, 1.36)	1.09 (0.87, 1.37)	1.17 (0.92, 1.48)
Maternal age				
20–24 years old		1.52** (1.18, 1.95)	1.43** (1.11, 1.85)	1.45** (1.12, 1.88)
25–29 years old		1.18 (0.94, 1.49)	1.14 (0.90, 1.44)	1.16 (0.91, 1.47)
30–34 years old		1.03 (0.81, 1.30)	1.00 (0.79, 1.27)	1.02 (0.80, 1.29)
35+ years old (Ref.)		1.00	1.00	1.00
Income				
$0–$22,000		0.76 (0.55, 1.04)	0.77 (0.56, 1.05)	0.82 (0.59, 1.12)
$22,001-37,000		1.07 (0.79, 1.46)	1.08 (0.80, 1.47)	1.08 (0.79, 1.46)
$37,001-52,000		1.10 (0.80, 1.50)	1.10 (0.80, 1.51)	1.11 (0.81, 1.52)
$52,001-67,000		0.89 (0.64, 1.24)	0.91 (0.65, 1.26)	0.92 (0.66, 1.28)
$67,001+ (Ref.)		1.00	1.00	1.00
Marital status				
Married		1.39** (1.17, 1.64)	1.38** (1.17, 1.64)	1.29** (1.09, 1.54)
Not married (Ref.)		1.00	1.00	1.00
Years of maternal education				
0–11 years		0.25** (0.18, 0.34)	0.26** (0.19, 0.35)	0.33** (0.24, 0.45)
12 years		0.26** (0.20, 0.33)	0.26** (0.20, 0.33)	0.30** (0.23, 0.39)
13–15 years		0.48** (0.38, 0.61)	0.49** (0.39, 0.62)	0.53** (0.41, 0.67)
16+ years (Ref.)		1.00	1.00	1.00
Race/ethnicity				
NH White (Ref.)		1.00	1.00	1.00
Hispanic		2.87** (2.21, 3.73)	2.82** (2.17, 3.67)	2.33** (1.79, 3.05)
NH Black		1.36 (0.98, 1.89)	1.38 (0.99, 1.92)	1.22 (0.87, 1.71)
NH Native American		2.98** (1.97, 4.53)	3.00** (1.97, 4.55)	2.56** (1.68, 3.92)
NH Other		3.25** (1.97, 5.36)	3.20** (1.94, 5.27)	3.17** (1.92, 5.25)
NH Asian		2.83** (1.60, 5.01)	2.85** (1.61, 5.03)	2.50** (1.41, 4.42)
Unknown race/ethnicity		1.70 (0.92, 3.12)	1.74 (0.94, 3.21)	1.51 (0.82, 2.79)
Insurance type				
Private insurance (Ref.)		1.00	1.00	1.00
Government insurance		0.75* (0.60, 0.93)	0.75* (0.60, 0.94)	0.81 (0.65, 1.01)
Other insurance		0.36** (0.23, 0.58)	0.37** (0.24, 0.59)	0.38** (0.24, 0.60)
No insurance		1.18 (0.90, 1.56)	1.18 (0.90, 1.55)	1.18 (0.89, 1.55)
HCW talked about breastfeeding before birth		1.70** (1.41, 2.05)	1.69** (1.40, 2.03)	1.68** (1.39, 2.03)
No (Ref.)		1.00	1.00	1.00
On WIC during pregnancy		0.73** (0.61, 0.88)	0.73** (0.61, 0.88)	0.73** (0.60, 0.88)
No (Ref.)			1.00	1.00
Experienced postpartum depression			0.81 (0.66, 1.00)	0.82 (0.67, 1.01)
No (Ref.)			1.00	1.00
Number of stresses in 12 months before birth			1.02 (0.98, 1.06)	1.05* (1.01, 1.09)
Wanted to be pregnant				
Then (Ref.)			1.00	1.00
Later			1.12 (0.92, 1.37)	1.15 (0.94, 1.41)
Sooner			1.04 (0.82, 1.31)	1.03 (0.81, 1.30)
Never			0.75* (0.57, 0.98)	0.77 (0.58, 1.01)
Unsure			0.90 (0.73, 1.11)	0.96 (0.78, 1.19)
Vaginal delivery (Ref.)				1.00
No				0.79** (0.67, 0.94)
Kotelchuck Index				
Inadequate				0.78* (0.61, 0.99)
Intermediate				0.77* (0.61, 0.98)
Adequate				1.04 (0.88, 1.24)
Adequate Plus (Ref.)				1.00
Hospital baby stay length				
0–2 days (Ref.)				1.00
3–5 days				1.08 (0.91, 1.30)
6+ days				1.45** (1.15, 1.82)
Smoking at time of survey				0.43** (0.36, 0.52)
No (Ref.)				1.00

*Note. CI* confidence interval, *HCW* healthcare worker, *NH* non-Hispanic, *OR* odds ratio, *WIC* Special Supplemental Nutrition Program for Women, Infants, and Children; *P < 0.05, **P < 0.01. The sample size is *N* = 10,159

Compared to non-Hispanic White women, those identifying as Hispanic, non-Hispanic Native American, non-Hispanic Asian, or non-Hispanic of a race not otherwise classified were more than twice as likely to initiate breastfeeding. Women with insurance other than private or government-sponsored were less likely to have ever breastfed compared to women with private insurance (OR 0.38; 95% CI 0.24, 0.60). Women who reported that a healthcare worker discussed breastfeeding before birth were over 1.5 times more likely to initiate breastfeeding than those who did not experience such conversations (OR 1.68; 95% CI 1.39, 2.03). Notably, prenatal WIC recipients were 27% less likely to have ever breastfed (OR 0.73; 95% CI 0.60, 0.88) compared to those who did not receive WIC.

With the exception of postpartum depression and pregnancy intention, all psychosocial and physiological variables under study were significantly associated with breastfeeding initiation in the final model. For example, with each additional stress a woman experienced in the 12 months prior to birth, the odds of breastfeeding initiation increased by 5% (OR 1.05; 95% CI 1.01, 1.09). The adequacy of prenatal care during pregnancy was also associated with breastfeeding initiation. Women who received intermediate and inadequate prenatal care were 22 -23% less likely to start breastfeeding compared to women who received adequate plus care. Further, a longer than typical newborn hospital stays (6 + days) was associated with an increased odd of breastfeeding initiation (OR 1.45; 95% CI 1.15, 1.82), whereas a non-vaginal delivery decreased the odds of breastfeeding initiation (OR 0.79; 95% CI 0.67, 0.94). Finally, smokers were more than 50% less likely to initiate breastfeeding (OR 0.43; 95% CI 0.36, 0.52) compared to non-smokers.

### Early breastfeeding cessation

Prenatal food insecurity was significantly related to early breastfeeding cessation in the unadjusted model (Table 4). Women who were food insecure in the 12 months prior to birth were more likely to breastfeed for < 1 week than to breastfeed for > 10 weeks compared to women who were food secure (RRR 2.22; 95% CI 1.55, 3.18). In other words, the risk of breastfeeding for < 1 week compared to breastfeeding for > 10 weeks more than doubled when prenatal food insecurity was reported. Similarly, women who were food insecure were more likely to stop breastfeeding at 1–3 weeks and 7–9 weeks compared to breastfeeding for > 10 weeks (Table 4). Although women who were food insecure also showed a higher risk of breastfeeding cessation at 4–6 weeks compared to > 10 weeks, this finding was not significant at the *P* < 0.01 level (to account for familywise error).

**Table 4:** Multinomial logistic regression using “breastfeeding for > 10 weeks” as reference category for Model 1

	Breastfeeding < 1 week, RRR (95% CI)	Breastfeeding 1–3 weeks, RRR (95% CI)	Breastfeeding 4–6 weeks, RRR (95% CI)	Breastfeeding 7–9 weeks, RRR (95% CI)
Food secure (Ref.)	1.00	1.00	1.00	1.00
Food insecure	2.22** (1.55, 3.18)	1.90** (1.51, 2.38)	1.36* (1.07, 1.72)	2.00** (1.59, 2.52)

*Note. CI* confidence interval, *RRR* relative risk ratio; *P < 0.05, **P < 0.01

When the sociodemographic potentially confounding variables were introduced in Model 2, the relationship between prenatal food insecurity and early breastfeeding cessation was no longer significant (Additional file 1). Likewise, when adding psychosocial variables into Model 3, no significant relationships between prenatal food insecurity and early breastfeeding cessation were noted at the *P* < 0.01 level (Additional file 2). However, as Table 5 indicates, when the physiological variables are included, the risk of breastfeeding for 4–6 weeks compared to breastfeeding for > 10 weeks was about 35% less for food insecure women relative to food secure women (RRR 0.65; 95% CI 0.50, 0.85; *P* < 0.01), though no other early cessation time frames achieved significance.

**Table 5:** Multinomial logistic regression using “breastfeeding for > 10 weeks” as reference category for Model 4

	Breastfeeding < 1 week, RRR (95% CI)	Breastfeeding 1–3 weeks, RRR (95% CI)	Breastfeeding 4–6 weeks, RRR (95% CI)	Breastfeeding 7–9 weeks, RRR (95% CI)
Food insecurity				
Food secure (Ref.)	1.00	1.00	1.00	1.00
Food insecure	0.92 (0.61, 1.37)	0.93 (0.72, 1.20)	0.65** (0.50, 0.85)	1.03 (0.79, 1.34)
Maternal age				
20–24 years old	0.95 (0.60, 1.48)	1.64** (1.23, 2.20)	1.36* (1.04, 1.77)	1.21 (0.91, 1.62)
25–29 years old	0.69 (0.45, 1.07)	1.25 (0.95, 1.64)	1.04 (0.82, 1.33)	0.97 (0.74, 1.28)
30–34 years old	0.65 (0.41, 1.03)	1.03 (0.78, 1.37)	0.85 (0.66, 1.09)	0.87 (0.66, 1.14)
35+ years old (Ref.)	1.00	1.00	1.00	1.00
Income				
$0–$22,000	1.40 (0.76, 2.58)	1.21 (0.86, 1.72)	1.14 (0.83, 1.57)	1.53* (1.08, 2.17)
$22,001-37,000	0.95 (0.52, 1.73)	1.00 (0.72, 1.40)	0.89 (0.66, 1.20)	1.31 (0.95, 1.82)
$37,001-52,000	1.35 (0.75, 2.42)	1.13 (0.81, 1.56)	0.96 (0.71, 1.30)	1.14 (0.81, 1.59)
$52,001-67,000	0.95 (0.47, 1.93)	1.17 (0.83, 1.67)	1.01 (0.73, 1.39)	0.90 (0.61, 1.33)
$67,001+ (Ref.)	1.00	1.00	1.00	1.00
Marital status				
Married	0.70* (0.51, 0.96)	0.78** (0.64, 0.94)	0.83* (0.69, 0.99)	0.77** (0.63, 0.94)
Not married (Ref.)	1.00	1.00	1.00	1.00
Years of maternal education				
0–11 years	3.71** (1.98, 6.96)	2.73** (1.93, 3.85)	2.79** (2.01, 3.88)	2.11** (1.48, 3.02)
12 years	5.18** (3.08, 8.70)	3.03** (2.29, 4.00)	3.36** (2.60, 4.34)	2.53** (1.91, 3.35)
13–15 years	3.14** (1.92, 5.13)	2.14** (1.66, 2.76)	2.28** (1.81, 2.87)	2.13** (1.66, 2.74)
16+ years (Ref.)	1.00	1.00	1.00	1.00
Race/ethnicity				
NH White (Ref.)	1.00	1.00	1.00	1.00
Hispanic	0.61* (0.39, 0.96)	0.95 (0.74, 1.23)	1.00 (0.78, 1.26)	1.05 (0.81, 1.35)
NH Black	0.46 (0.21, 1.00)	0.81 (0.54, 1.21)	1.01 (0.70, 1.44)	1.02 (0.69, 1.50)
NH Native American	0.97 (0.56, 1.68)	0.87 (0.59, 1.28)	0.75 (0.50, 1.12)	0.91 (0.61, 1.35)
NH Other	0.52 (0.26, 1.04)	0.66* (0.44, 0.98)	0.54** (0.35, 0.82)	0.65* (0.42, 0.99)
NH Asian	0.27 (0.07, 1.10)	0.79 (0.48, 1.31)	1.11 (0.75, 1.66)	1.24 (0.81, 1.91)
Unknown Race	0.62 (0.19, 2.02)	0.63 (0.31, 1.27)	0.87 (0.49, 1.55)	0.48 (0.21, 1.11)
Insurance type				
Private insurance (Ref.)	1.00	1.00	1.00	1.00
Government insurance	1.13 (0.76, 1.66)	1.00 (0.79, 1.26)	0.86 (0.68, 1.07)	1.03 (0.80, 1.31)
Other insurance	1.05 (0.31, 3.53)	1.46 (0.80, 2.69)	0.79 (0.40, 1.60)	1.11 (0.55, 2.22)
No insurance	0.79 (0.49, 1.27)	0.84 (0.64, 1.12)	0.72* (0.55, 0.95)	1.09 (0.82, 1.43)
HCW talked about breastfeeding before birth	0.75 (0.52, 1.09)	1.39* (1.07, 1.81)	1.00 (0.80, 1.24)	1.42* (1.09, 1.85)
No (Ref.)	1.00	1.00	1.00	11.00
On WIC during pregnancy	1.43* (1.01, 2.02)	1.23 (1.00, 1.51)	1.27* (1.04, 1.55)	0.95 (0.77, 1.17)
No (Ref.)	1.00	1.00	1.00	11.00
Experienced postpartum depression	0.93 (0.61, 1.41)	1.50** (1.19, 1.89)	1.32* (1.05, 1.65)	1.14 (0.89, 1.47)
No (Ref.)	1.00	1.00	1.00	11.00
Number of stresses in 12 months before birth	1.00 (0.93, 1.07)	0.97 (0.93, 1.01)	1.02 (0.98, 1.06)	1.00 (0.96, 1.05)
Wanted to be pregnant				
Then (Ref.)	1.00	1.00	1.00	1.00
Later	1.24 (0.86, 1.78)	1.10 (0.89, 1.35)	0.99 (0.80, 1.22)	1.08 (0.87, 1.34)
Sooner	1.09 (0.69, 1.72)	0.99 (0.77, 1.28)	1.16 (0.92, 1.45)	0.92 (0.71, 1.20)
Never	1.99** (1.25, 3.16)	0.83 (0.58, 1.19)	1.05 (0.77, 1.45)	0.73 (0.49, 1.06)
Unsure	1.10 (0.73, 1.66)	1.04 (0.81, 1.33)	1.15 (0.91, 1.45)	1.02 (0.79, 1.31)
Vaginal delivery (Ref.)	1.00	1.00	1.00	1.00
No	1.31 (0.96, 1.77)	1.60** (1.34, 1.92)	1.27** (1.07, 1.51)	1.10 (0.91, 1.33)
Kotelchuck Index				
Inadequate	0.89 (0.56, 1.41)	1.05 (0.81, 1.37)	0.83 (0.64, 1.08)	1.05 (0.79, 1.38)
Intermediate	0.94 (0.60, 1.48)	0.93 (0.71, 1.21)	0.90 (0.70, 1.16)	0.98 (0.74, 1.30)
Adequate	0.88 (0.65, 1.20)	0.73** (0.61, 0.88)	0.70** (0.59, 0.84)	0.97 (0.80, 1.17)
Adequate Plus (Ref.)	1.00	1.00	1.00	1.00
Hospital baby stay length				
0–2 days (Ref.)	1.00	1.00	1.00	1.00
3–5 days	1.09 (0.79, 1.50)	1.03 (0.85, 1.26)	1.24* (1.03, 1.50)	1.16 (0.94, 1.44)
6 + days	0.73 (0.46, 1.14)	1.14 (0.89, 1.45)	1.84** (1.49, 2.27)	2.12** (1.69, 2.66)
Smoking at time of survey	2.59** (1.88, 3.57)	2.74** (2.22, 3.38)	2.46** (2.01, 3.02)	2.12** (1.69, 2.65)
No (Ref.)	1.00	1.00	1.00	1.00

*Note. CI* confidence interval, *HCW* healthcare worker, *NH* non-Hispanic, *RRR* relative risk ratio, *WIC* Special Supplemental Nutrition Program for Women, Infants, and Children; *P < 0.05, **P < 0.01. The sample size is *N* = 9239

Women aged 20–24 years old had an increased risk of breastfeeding cessation at 1–3 weeks versus > 10 weeks compared to women 35 years and older, though no other age ranges or cessation periods showed significance. Among married women, the risk of breastfeeding cessation at 1–3 weeks and 7–9 weeks versus > 10 weeks was between 22 and 23% lower compared to unmarried women. Additionally, women with less years of education were more likely to discontinue breastfeeding earlier. In fact, women with < 16 years of education had more than a 3–5 fold greater risk of breastfeeding for < 1 week versus > 10 weeks compared to women with > 16 years of education. Racial/ethnic identity was not associated with early breastfeeding cessation, except among non-Hispanic women of a race not otherwise classified, who exhibited a 46% lower risk of breastfeeding cessation at 4–6 weeks versus > 10 weeks compared to non-Hispanic White women. No other sociodemographic variables (i.e., income, insurance type at the time of survey, breastfeeding information provided by healthcare provider, and WIC status during pregnancy) were significantly related to early breastfeeding cessation at the *P* < 0.01 level.

Psychosocially, women who experienced postpartum depression had a greater risk of breastfeeding cessation at 1–3 weeks (RRR 1.50; 95% CI 1.19, 1.89) than to breastfeed for > 10 weeks. Compared to those who wanted to be pregnant at the time of their pregnancy, women who never wanted to be pregnant had a 2-fold higher risk of breastfeeding for < 1 week than > 10 weeks (RRR 1.99; 95% CI 1.25, 3.16). The number of stresses in the 12 months prior to birth was not significant for any time interval.

Among the physiological variables, women who did not deliver vaginally had significantly higher risk of breastfeeding cessation at 1–3 weeks (RRR 1.60; 95% CI 1.34, 1.92) and 4–6 weeks (RRR 1.27; 95% CI 1.07, 1.51) than to breastfeed for > 10 weeks. In addition, women whose infants remained in the hospital for > 6 days were 84 -112% more likely to stop breastfeeding at 4–6 weeks or 7–9 weeks than to breastfeed for > 10 weeks when compared to women whose infants stayed in the hospital for 0–2 days. With regards to prenatal care, those receiving adequate care had a lower risk of breastfeeding cessation at 1–3 weeks (RRR 0.73; 95% CI 0.61, 0.88) and 4–6 weeks (RRR 0.70; 95% CI 0.59, 0.84) versus > 10 weeks, compared to women receiving adequate plus prenatal care. Finally, women who smoked at the time of the survey had more than a two-fold greater risk of discontinuing breastfeeding at all shorter time intervals than to breastfeed for > 10 weeks, compared to non-smokers (Table 5).

## Discussion

Ninety percent of women in this study were food secure prior to the birth of their infants and/or initiated breastfeeding, and more than half continued breastfeeding for > 10 weeks regardless of their food security status. Among this sample, food insecurity was not associated with breastfeeding initiation after adjusting for socioeconomic, psychosocial, and physiological factors. This finding is congruent with results from a similar study by Orr et al. [23], who found that among 10,450 Canadian women, breastfeeding initiation was unrelated to household food security once sociodemographic characteristics were taken into account. Conversely, in a qualitative study of 20 food insecure Nova Scotian mothers by Frank [24], 95% initiated breastfeeding due to the health benefits and high cost of formula. Frank states that “worry over the cost of formula was a driving factor in initiation, indicating that household food insecurity could be a predictor of breastfeeding initiation” [24]. However, this sample was drawn from the federally funded community-based projects of the Canada Prenatal Nutrition Programs (CPNP), which work to improve maternal and infant health though breastfeeding promotion and other types of support [41]. The high breastfeeding initiation rates in this sample of food insecure mothers may be influenced by CPNP participation [24].

Likewise, prenatal food security status was not related to three of the four early breastfeeding cessation time frames we analyzed. Our findings support the results from Gomes and Gubert’s [22] nationally representative study in Brazil that show no significant relationship between food insecurity and any breastfeeding among children younger than 12 months. It should be noted, though, that the researchers did not account for sociodemographic variables known to be associated with breastfeeding and food security, such as maternal education level, marital status, employment, maternal health factors, etc. [22]. However, our findings differ from the qualitative results from Haiti [21] and Nova Scotia [24], which suggest that food insecurity leads to breastfeeding cessation due to maternal weakness, perceived or actual breast milk insufficiency, and/or concern that breastmilk is not nutritionally adequate because of poor maternal diet. Other studies that have measured exclusive breastfeeding show either no relationship with hunger [18, 19, 26, 27], or an association between food insecurity and increased likelihood of earlier exclusive breastfeeding cessation [19, 23].

Unfortunately, our data do not clearly explain why the risk of breastfeeding cessation at 4–6 weeks compared to breastfeeding for > 10 weeks is significantly lower when food insecurity is experienced prenatally, independent of other known influences on breastfeeding. It may be a result of a relatively small number (*n* = 87) of women who reported both food insecurity and breastfeeding for 4–6 weeks, as no other breastfeeding cessation timeframe was significantly different from > 10 weeks when covariates were controlled. Additionally, whereas the percentage of food insecure women who stopped breastfeeding at 4–6 weeks was lower than either 1–3 weeks or 7–9 weeks, the percentage of food secure women who stopped breastfeeding was higher at 4–6 weeks than either 1–3 weeks and 7–9 weeks. These differences in cessation patterns by food security status may have led to the finding that food insecurity appears protective for early breastfeeding cessation at 4–6 weeks. An alternative explanation may be that non-working or underemployed mothers with low socioeconomic status may breastfeed longer due to increased proximity to their infants coupled with the high cost of formula feeding, of up to $1500 for a year [42]. Yet reports from food-insecure mothers in Canada indicate that breastfeeding does not save money because there is often little money for food to begin with, regardless of the infant feeding method [24]. Further qualitative research is needed in the U.S. to explore the factors driving this association.

Our findings regarding other breastfeeding predictors mirror prior work that suggests initiation and longer durations are related to a variety of sociodemographic, psychosocial, and physiological characteristics [4, 20, 43, 44]. Specifically, we found that married and college-educated women, non-smokers, women not receiving WIC during pregnancy, and women who had vaginal deliveries were more likely to initiate breastfeeding and/or breastfeed for longer, regardless of food security status. Further public health and policy efforts to improve breastfeeding initiation and duration rates should therefore concentrate on single mothers, those without a college degree, smokers, prenatal WIC recipients, and those who have non-vaginal births. For example, smokers often choose to formula feed because they fear their breast milk is contaminated by smoking and thus harmful to the baby [45]. Yet the CDC states that although mothers should be encouraged to quit tobacco and e-cigarette smoking, breastfeeding is not contraindicated among mothers who smoke but instead remains the recommended food for an infant [46]. While pregnancy and lactation are opportune times for promoting smoking cessation, healthcare professionals should educate mothers that they can continue to breastfeed whether or not they are able to quit.

It is notable that participation in WIC during pregnancy decreased the odds of breastfeeding initiation given the measures WIC has put in place to support breastfeeding. In 1997, WIC launched the *Loving Support Makes Breastfeeding Work* campaign to increase initiation and duration rates among WIC participants and raise public acceptance and breastfeeding support through mass media campaigns, participant education materials, and technical assistance for WIC staff [47]. Additionally, WIC participants who breastfeed receive a greater quantity and variety of foods than non-breastfeeding participants, along with breast pumps, one-on-one support from WIC Peer Counselors, and longer participation in the program [47]. Still, not all mothers are aware of WIC’s breastfeeding resources, and not all WIC agencies have delivered the campaign consistently [48]. Likewise, while the benefits of breastfeeding are known, WIC participants lack breastfeeding skills and confidence and find breastfeeding to be more difficult than expected [48]. WIC provides free infant formula (coupled with less food) to mothers who are unable or choose not to breastfeed, and WIC’s budget for breastfeeding promotion is small in comparison to the amount spent on obtaining infant formula [49]. Critics suggest that WIC’s provision of no-cost infant formula is both an implicit endorsement of formula by the U.S. government and an incentive for use by WIC participants [49]. Calls to phase out WIC’s large-scale formula distribution [49] have not been answered to date, though WIC launched a new breastfeeding promotion and support campaign (*Learn Together. Grow Together.*) in 2018 to address the awareness and implementation issues of the prior campaign [48]. Although WIC cannot be expected to address all of the social determinants of breastfeeding behaviors, future research is needed to determine the extent to which WIC’s updated campaign is successful in increasing recipients’ breastfeeding skills, confidence, and utilization of resources.

This study is not without limitations that should be mentioned. First, a more diverse sample by race, ethnicity, education level, food insecurity, and breastfeeding initiation may show stronger associations between the variables of interest. As the data were taken from a small selection of states that mostly exhibit higher-than-average rates of breastfeeding initiation and household food security, these results may not be generalizable to all food insecure women in other regions of the U.S. or internationally. We recommend that more states include food security questions in their PRAMS questionnaires, or that food security questions become part of the core PRAMS survey. Similarly, it is possible that food insecure populations may not participate in national surveys like PRAMS due to high mobility and a possible lack of consistent phone coverage. This represents a potential selection bias that must be considered when interpreting our results.

Logistic regressions typically require a large sample size and a general guideline is that a minimum of 10 cases is needed for the least frequent outcome of each independent variable in the model [50]. As such, we checked for small cells by performing a cross-tabulation between categorical predictors and breastfeeding cessation. There were a few cases where the cell size did fall below the threshold of 10. All but one of these instances occurred in the race categories. For example, among women who breastfed for < 1 week, there were only seven non-Hispanic Black women, nine non-Hispanic women of a race not otherwise classified, two non-Hispanic Asian women, and three women of an unknown race and ethnicity. Likewise, among women of an unknown race and ethnicity, there were nine who breastfed for 1–3 weeks and six who breastfeed for 7–9 weeks. One other small cell occurrence was noted for the three participants with other insurance who breastfed for < 1 week. Since these small cells have the potential to make our model unstable, we re-ran our models with larger time-based intervals: < 4 weeks, 5–9 weeks, and > 10 weeks. The results from these multinomial logistic regressions were similar to those of our original models included in this study. Unfortunately, these larger time-based intervals are not useful for policy recommendations as they do not represent meaningful postpartum time frames (i.e., 6-week maternity leave). Although the use of weighted variables can potentially reduce accuracy because the sampling variance, standard deviation, and standard error increase, it is important to note that the data were weighted and are representative of the 12-month PRAMS-eligible population. As a result, our weighted sample is representative of the women age 20 years or older who gave birth to live infants in the selected states.

Additionally, food security status was determined from a single question, which may not provide the sensitivity needed to identify all women experiencing food insecurity nor capture the latent construct as precisely as the standard 18-item or abbreviated 6-item food security modules used by the USDA [32]. More specifically, the food security question asked in PRAMS reflects reduced adult food intake, and while this alone cannot capture the full range of food insecurity, it is asking for a level of severity that goes beyond anxiety about food budgets or food supply. There are no studies, to our knowledge, that examine food insecurity and breastfeeding using a 1-item measure for food insecurity, however other studies examining food insecurity using the PRAMS dataset do use the 1-item measure [33, 34]. Future PRAMS surveys would benefit from including additional validated food security questions to better discern between various levels of food security.

To understand how food insecurity, as measured, can impact a distal outcome such as breastfeeding beyond 10 weeks, it is important to understand the nature of food insecurity. Food insecurity is rarely an acute problem, but rather a prolonged and chronic issue that actively occurs over extended periods of time [14]. Although we only capture a snapshot of food security status, we can safely assume that some respondents may be food insecure for periods longer than 12 months. Due to the limitations in this question we cannot ascertain the exact time period, but this measure may not be as distal as it seems on the surface. Likewise, PRAMS does not include a measure of current food security status at the time of the survey, so it remains unclear if women are food insecure while breastfeeding. Finally, there are variables not included in PRAMS that are related to food insecurity and/or breastfeeding, such as employment status at the time of the survey, percent of the federal poverty level, and postnatal participation in WIC or other federal food assistance programs. Including these questions in future PRAMS questionnaires would allow for testing alternative explanations for the mainly null associations we found between food security and breastfeeding outcomes.

## Conclusions

Although PRAMS is a cross-sectional survey, the results of this study add to the small body of international literature on food insecurity and breastfeeding outcomes. Among this U.S. sample, socioeconomic, psychosocial, and physiological factors explain the association between prenatal food insecurity and breastfeeding outcomes. Our findings indicate the need for more targeted and effective interventions and policies in the U.S. that encourage the initiation and duration of breastfeeding regardless of food security status, particularly among single mothers, those without a college degree, smokers, prenatal WIC recipients, and those recovering from non-vaginal births.

## Supplementary information

**Additional file 1. **Table of multinomial logistic regression using “breastfeeding for > 10 weeks” as reference category for Model 2: Pregnancy Risk Assessment Monitoring System, Colorado, Maine, New Mexico, Oregon, Pennsylvania, and Vermont, 2012–2013.

**Additional file 2. **Table of multinomial logistic regression using “breastfeeding for > 10 weeks” as reference category for Model 3: Pregnancy Risk Assessment Monitoring System, Colorado, Maine, New Mexico, Oregon, Pennsylvania, and Vermont, 2012–2013.

## Data Availability

The data that support the findings of this study are available from the United States Centers for Disease Control and Prevention (CDC), but restrictions apply to the availability of these data, which were used under license for the current study, and so the authors are unable to release the data set. Data are however available from the CDC via a proposal submission process outlined here: https://www.cdc.gov/prams/prams-data/researchers.htm. The survey questionnaires, analytic variables, and codebooks are publicly available from the PRAMS website: https://www.cdc.gov/prams/.

## Abbreviations

CDC: Centers for Disease Control and Prevention
CHIP: Children’s Health Insurance Program
CI: Confidence interval
CPNP: Canada Prenatal Nutrition Programs
OR: Odds ratio
PRAMS: Pregnancy Risk Assessment Monitoring System
RRR: Relative risk ratio
USDA: United States Department of Agriculture
WIC: Special Supplemental Nutrition Program for Women, Infants, and Children
