# Supplementary material for: The role of prenatal food insecurity on breastfeeding behaviors: findings from the United States pregnancy risk assessment monitoring system
Source: Int Breastfeed J. 2020 Apr 19;15:30. doi: 10.1186/s13006-020-00276-x (PMC7169030; doi:10.1186/s13006-020-00276-x)
Supplement: Supplementary file 1 — Additional file 1. Table of multinomial logistic regression using “breastfeeding for > 10 weeks” as reference category for Model 2: Pregnancy Risk Assessment Monitoring System, Colorado, Maine, New Mexico, Oregon, Pennsylvania, and Vermont, 2012–2013. [file 13006_2020_276_MOESM1_ESM.docx]

**Additional File 1.** Multinomial logistic regression using “breastfeeding for >10 weeks” as reference category for Model 2: Pregnancy Risk Assessment Monitoring System, Colorado, Maine, New Mexico, Oregon, Pennsylvania, and Vermont, 2012-2013.

|  | **Breastfeeding**  **<1 week, RRR**  **(95% CI)** | **Breastfeeding**  **1-3 weeks, RRR**  **(95% CI)** | **Breastfeeding**  **4-6 weeks, RRR**  **(95% CI)** | **Breastfeeding**  **7-9 weeks, RRR**  **(95% CI)** |
| --- | --- | --- | --- | --- |
| Food insecurity |  |  |  |  |
| Food secure (Ref.) | 1.00 | 1.00 | 1.00 | 1.00 |
| Food insecure | 1.07 (0.74, 1.56) | 1.08 (0.85, 1.38) | 0.81 (0.63, 1.05) | 1.17 (0.92, 1.50) |
| Maternal age |  |  |  |  |
| 20-24 years old | 0.87 (0.57, 1.35) | 1.52** (1.14, 2.01) | 1.21 (0.93, 1.55) | 1.21 (0.91, 1.59) |
| 25-29 years old | 0.65* (0.42, 0.99) | 1.18 (0.90, 1.54) | 0.95 (0.75, 1.21) | 0.97 (0.75, 1.27) |
| 30-34 years old | 0.63* (0.40, 0.98) | 1.00 (0.76, 1.32) | 0.82 (0.64, 1.04) | 0.87 (0.67, 1.15) |
| 35+ years old (Ref.) | 1.00 | 1.00 | 1.00 | 1.00 |
| Income |  |  |  |  |
| $0-$22,000 | 1.58 (0.86, 2.90) | 1.32 (0.94, 1.86) | 1.27 (0.93, 1.73) | 1.62** (1.15, 2.29) |
| $22,001-37,000 | 0.99 (0.54, 1.80) | 1.01 (0.73, 1.40) | 0.92 (0.68, 1.24) | 1.34 (0.97, 1.84) |
| $37,001-52,000 | 1.41 (0.79, 2.53) | 1.15 (0.83, 1.59) | 1.00 (0.74, 1.34) | 1.15 (0.83, 1.61) |
| $52,001-67,000 | 1.01 (0.50, 2.02) | 1.20 (0.85, 1.69) | 1.02 (0.75, 1.40) | 0.91 (0.62, 1.33) |
| $67,001+ (Ref.) | 1.00 | 1.00 | 1.00 | 1.00 |
| Marital status |  |  |  |  |
| Married | 0.62** (0.46, 0.84) | 0.71** (0.59, 0.86) | 0.75** (0.63, 0.90) | 0.71** (0.59, 0.86) |
| Not married (Ref.) | 1.00 | 1.00 | 1.00 | 1.00 |
| Years of maternal education |  |  |  |  |
| 0-11 years | 4.44** (2.39, 8.24) | 3.35** (2.40, 4.68) | 3.29** (2.39, 4.53) | 2.34** (1.65, 3.33) |
| 12 years | 5.94** (3.57, 9.88) | 3.48** (2.66, 4.57) | 3.82** (2.97, 4.90) | 2.75** (2.08, 3.63) |
| 13-15 years | 3.42** (2.10, 5.57) | 2.26** (1.76, 2.89) | 2.39** (1.90, 2.99) | 2.19** (1.70, 2.80) |
| 16+ years (Ref.) | 1.00 | 1.00 | 1.00 | 1.00 |
| Race/ethnicity |  |  |  |  |
| NH White (Ref.) | 1.00 | 1.00 | 1.00 | 1.00 |
| Hispanic | 0.49** (0.32, 0.77) | 0.77* (0.60, 0.98) | 0.78* (0.62, 0.99) | 0.89 (0.69, 1.14) |
| NH Black | 0.41* (0.19, 0.89) | 0.72 (0.48, 1.07) | 0.90 (0.63, 1.28) | 0.94 (0.64, 1.37) |
| NH Native American | 0.77 (0.45, 1.32) | 0.69 (0.47, 1.00) | 0.56** (0.37, 0.83) | 0.73 (0.49, 1.07) |
| NH Other | 0.52 (0.26, 1.04) | 0.66* (0.44, 0.97) | 0.51** (0.34, 0.77) | 0.63* (0.41, 0.96) |
| NH Asian | 0.24* (0.06, 0.97) | 0.69 (0.42, 1.14) | 0.95 (0.64, 1.42) | 1.09 (0.72, 1.67) |
| Unknown Race | 0.55 (0.17, 1.77) | 0.60 (0.30, 1.20) | 0.82 (0.46, 1.46) | 0.46 (0.20, 1.07) |
| Insurance type |  |  |  |  |
| Private insurance (Ref.) | 1.00 | 1.00 | 1.00 | 1.00 |
| Government insurance | 1.22 (0.83, 1.80) | 1.11 (0.88, 1.40) | 0.98 (0.78, 1.22) | 1.13 (0.89, 1.44) |
| Other insurance | 1.07 (0.32, 3.58) | 1.51 (0.83, 2.73) | 0.79 (0.40, 1.57) | 1.10 (0.55, 2.20) |
| No insurance | 0.77 (0.47, 1.23) | 0.82 (0.62, 1.08) | 0.69** (0.53, 0.90) | 1.06 (0.81, 1.40) |
| HCW talked about breastfeeding before birth | 0.74 (0.51, 1.06) | 1.34* (1.04, 1.74) | 0.95 (0.76, 1.17) | 1.37* (1.05, 1.79) |
| No (Ref.) | 1.00 | 1.00 | 1.00 | 1.00 |
| On WIC during pregnancy | 1.47* (1.04, 2.07) | 1.23* (1.00, 1.51) | 1.28* (1.05, 1.56) | 0.94 (0.77, 1.16) |
| No (Ref.) | 1.00 | 1.00 | 1.00 | 1.00 |

*Note.* **CI** = confidence interval; **HCW** = healthcare worker; **NH** = non-Hispanic; **RRR** = relative risk ratio; **WIC** = Special Supplemental Nutrition Program for Women, Infants, and Children; *P<0.05, **P<0.01.
