# Supplementary material for: The role of prenatal food insecurity on breastfeeding behaviors: findings from the United States pregnancy risk assessment monitoring system
Source: Int Breastfeed J. 2020 Apr 19;15:30. doi: 10.1186/s13006-020-00276-x (PMC7169030; doi:10.1186/s13006-020-00276-x)
Supplement: Supplementary file 2 — Additional file 2. Table of multinomial logistic regression using “breastfeeding for > 10 weeks” as reference category for Model 3: Pregnancy Risk Assessment Monitoring System, Colorado, Maine, New Mexico, Oregon, Pennsylvania, and Vermont, 2012–2013. [file 13006_2020_276_MOESM2_ESM.docx]

**Additional File 2.** Multinomial logistic regression using “breastfeeding for >10 weeks” as reference category for Model 3: Pregnancy Risk Assessment Monitoring System, Colorado, Maine, New Mexico, Oregon, Pennsylvania, and Vermont, 2012-2013.

|  | **Breastfeeding**  **<1 week, RRR**  **(95% CI)** | **Breastfeeding**  **1-3 weeks, RRR**  **(95% CI)** | **Breastfeeding**  **4-6 weeks, RRR**  **(95% CI)** | **Breastfeeding**  **7-9 weeks, RRR**  **(95% CI)** |
| --- | --- | --- | --- | --- |
| Food insecurity |  |  |  |  |
| Food secure (Ref.) | 1.00 | 1.00 | 1.00 | 1.00 |
| Food insecure | 1.00 (0.67, 1.50) | 1.02 (0.79, 1.32) | 0.71* (0.54, 0.92) | 1.10 (0.85, 1.43) |
| Maternal age |  |  |  |  |
| 20-24 years old | 0.91 (0.58, 1.42) | 1.48** (1.11, 1.97) | 1.24 (0.96, 1.61) | 1.15 (0.87, 1.53) |
| 25-29 years old | 0.68 (0.44, 1.05) | 1.17 (0.89, 1.53) | 0.98 (0.77, 1.25) | 0.95 (0.73, 1.24) |
| 30-34 years old | 0.65 (0.41, 1.02) | 0.99 (0.75, 1.31) | 0.83 (0.65, 1.06) | 0.86 (0.66, 1.13) |
| 35+ years old (Ref.) | 1.00 | 1.00 | 1.00 | 1.00 |
| Income |  |  |  |  |
| $0-$22,000 | 1.45 (0.79, 2.68) | 1.29 (0.91, 1.82) | 1.19 (0.87, 1.63) | 1.58* (1.11, 2.23) |
| $22,001-37,000 | 0.93 (0.51, 1.70) | 1.00 (0.72, 1.39) | 0.89 (0.66, 1.20) | 1.32 (0.95, 1.83) |
| $37,001-52,000 | 1.34 (0.75, 2.41) | 1.14 (0.82, 1.57) | 0.97 (0.72, 1.31) | 1.14 (0.81, 1.59) |
| $52,001-67,000 | 0.96 (0.47, 1.93) | 1.18 (0.84, 1.67) | 1.01 (0.73, 1.38) | 0.90 (0.61, 1.32) |
| $67,001+ (Ref.) | 1.00 | 1.00 | 1.00 | 1.00 |
| Marital status |  |  |  |  |
| Married | 0.65** (0.48, 0.89) | 0.72** (0.59, 0.86) | 0.76** (0.64, 0.91) | 0.72** (0.59, 0.87) |
| Not married (Ref.) | 1.00 | 1.00 | 1.00 | 1.00 |
| Years of maternal education |  |  |  |  |
| 0-11 years | 4.44** (2.39, 8.27) | 3.33** (2.38, 4.66) | 3.26** (2.37, 4.50) | 2.39** (1.68, 3.39) |
| 12 years | 5.80** (3.48, 9.67) | 3.47** (2.64, 4.55) | 3.76** (2.93, 4.83) | 2.76** (2.09, 3.65) |
| 13-15 years | 3.33** (2.04, 5.43) | 2.25** (1.76, 2.89) | 2.36** (1.88, 2.96) | 2.20** (1.71, 2.81) |
| 16+ years (Ref.) | 1.00 | 1.00 | 1.00 | 1.00 |
| Race/ethnicity |  |  |  |  |
| NH White (Ref.) | 1.00 | 1.00 | 1.00 | 1.00 |
| Hispanic | 0.51** (0.32, 0.79) | 0.78* (0.61, 0.99) | 0.81 (0.64, 1.02) | 0.90 (0.70, 1.15) |
| NH Black | 0.39* (0.18, 0.86) | 0.71 (0.47, 1.05) | 0.89 (0.62, 1.26) | 0.93 (0.64, 1.37) |
| NH Native American | 0.79 (0.46, 1.36) | 0.68* (0.47, 0.99) | 0.56** (0.37, 0.83) | 0.73 (0.49, 1.08) |
| NH Other | 0.53 (0.26, 1.05) | 0.65* (0.44, 0.97) | 0.51** (0.33, 0.77) | 0.62* (0.41, 0.95) |
| NH Asian | 0.24* (0.06, 0.96) | 0.68 (0.41, 1.11) | 0.95 (0.64, 1.41) | 1.10 (0.72, 1.68) |
| Unknown Race | 0.57 (0.18, 1.83) | 0.58 (0.28, 1.16) | 0.81 (0.45, 1.44) | 0.47 (0.20, 1.08) |
| Insurance type |  |  |  |  |
| Private insurance (Ref.) | 1.00 | 1.00 | 1.00 | 1.00 |
| Government insurance | 1.20 (0.82, 1.76) | 1.10 (0.88, 1.39) | 0.95 (0.76, 1.19) | 1.12 (0.88, 1.43) |
| Other insurance | 1.09 (0.33, 3.62) | 1.49 (0.82, 2.71) | 0.78 (0.39, 1.55) | 1.11 (0.55, 2.20) |
| No insurance | 0.76 (0.47, 1.22) | 0.82 (0.62, 1.07) | 0.69** (0.53, 0.90) | 1.05 (0.80, 1.38) |
| HCW talked about breastfeeding before birth | 0.76 (0.52, 1.10) | 1.35* (1.04, 1.75) | 0.96 (0.78, 1.19) | 1.37* (1.05, 1.79) |
| No (Ref.) | 1.00 | 1.00 | 1.00 | 1.00 |
| On WIC during pregnancy | 1.47* (1.04, 2.06) | 1.22 (1.00, 1.50) | 1.27* (1.04, 1.55) | 0.94 (0.76, 1.15) |
| No (Ref.) | 1.00 | 1.00 | 1.00 | 1.00 |
| Experienced postpartum depression | 0.94 (0.62, 1.42) | 1.54** (1.23, 1.93) | 1.38** (1.11, 1.72) | 1.20 (0.93, 1.54) |
| No (Ref.) | 1.00 | 1.00 | 1.00 | 1.00 |
| Number of stresses in 12 months before birth | 1.02 (0.95, 1.09) | 1.00 (0.96, 1.04) | 1.04* (1.00, 1.09) | 1.02 (0.98, 1.07) |
| Wanted to be pregnant |  |  |  |  |
| Then (Ref.) | 1.00 | 1.00 | 1.00 | 1.00 |
| Later | 1.24 (0.87, 1.78) | 1.10 (0.89, 1.35) | 0.99 (0.80, 1.21) | 1.09 (0.88, 1.34) |
| Sooner | 1.11 (0.70, 1.75) | 1.02 (0.79, 1.31) | 1.21 (0.97, 1.51) | 0.95 (0.73, 1.24) |
| Never | 2.01** (1.27, 3.19) | 0.86 (0.60, 1.23) | 1.09 (0.80, 1.50) | 0.76 (0.52, 1.11) |
| Unsure | 1.17 (0.78, 1.76) | 1.11 (0.88, 1.42) | 1.21 (0.96, 1.51) | 1.05 (0.82, 1.35) |
|  |  |  |  |  |

*Note.* **CI** = confidence interval; **HCW** = healthcare worker; **NH** = non-Hispanic; **RRR** = relative risk ratio; **WIC** = Special Supplemental Nutrition Program for Women, Infants, and Children; *P<0.05, **P<0.01.
